# Grading recommendations for enhanced patient safety in sentinel event analysis: the recommendation improvement matrix

**DOI:** 10.1136/bmjoq-2023-002592

**Published:** 2024-04-16

**Authors:** Kelly Bos, Maarten J van der Laan, Jop Groeneweg, Gert Jan Kamps, Dink A Legemate, Ian Leistikow, Dave A Dongelmans

**Affiliations:** 1 Department of Surgery, University Medical Centre Groningen, Groningen, The Netherlands; 2 Delft University of Technology, TU Delft, Delft, The Netherlands; 3 University of Leiden, Leiden, The Netherlands; 4 Intergo International Centre for Safety Ergonomics and Human Factors, Amersfoort, The Netherlands; 5 Department of Surgery, Amsterdam UMC Location AMC, Amsterdam, The Netherlands; 6 Erasmus University Rotterdam, Rotterdam, The Netherlands; 7 Department of Intensive Care Medicine, Amsterdam UMC Locatie AMC, Amsterdam, The Netherlands; 8 Amsterdam Public Health Research Institute, Amsterdam, The Netherlands

**Keywords:** healthcare quality improvement, incident reporting, patient safety, quality improvement, safety management

## Abstract

**Objectives:**

The goal of sentinel event (SE) analysis is to prevent recurrence. However, the rate of SEs has remained constant over the past years. Research suggests this is in part due to the quality of recommendations. Currently, standards for the selection of recommendations are lacking. Developing a method to grade recommendations could help in both designing and selecting interventions most likely to improve patient safety. The aim of this study was to (1) develop a user-friendly method to grade recommendations and (2) assess its applicability in a large series of Dutch perioperative SE analysis reports.

**Methods:**

Based on two grading methods, we developed the recommendation improvement matrix (RIM). Applicability was assessed by analysing all Dutch perioperative SE reports over a 12-month period. After which interobserver agreement was studied.

**Results:**

In the RIM, two elements are crucial: whether the recommendation intervenes before or after an SE and whether it eliminates or controls the hazard. Applicability was evaluated in 115 analysis reports, encompassing 161 recommendations. Recommendation quality varied from the highest, category A, to the lowest, category D, with category A accounting for 44%, category B for 35%, category C for 2% and category D for 19% of recommendations. There was a fair interobserver agreement.

**Conclusion:**

The RIM can be used to grade recommendations in SE analysis and could possibly help in both designing and selecting interventions. It is relatively simple, user-friendly and has the potential to improve patient safety. The RIM can help formulate effective and sustainable recommendations, a second key objective of the RIM is to foster and facilitate constructive dialogue among those responsible for patient safety.

**WHAT IS ALREADY KNOWN ON THIS TOPIC**

- The field of healthcare currently lacks established standards or methods for grading recommendations following the analysis of sentinel events.

**WHAT THIS STUDY ADDS**

- This research introduces the recommendation improvement matrix (RIM), a user-friendly and innovative method for grading recommendations after sentinel events.

**HOW THIS STUDY MIGHT AFFECT RESEARCH, PRACTICE OR POLICY**

- The RIM offers essential insights that foster constructive discussions about recommendations postsentinel events among professionals responsible for patient safety.
- The implementation of RIM could significantly contribute to safer healthcare practices by improving the quality of recommendations and ensuring their practical applicability.

## Introduction

The primary goal of the analysis of a sentinel event (SE) in healthcare is to prevent the recurrence of similar events in the future. Really improving patient safety on the basis of these analysis has been proven challenging.^1^ The frequency of SEs, which are incidents that lead to death or serious harm to patients, has essentially remained unchanged in recent years.^1 2^ Improving patient safety after an SE is based on a learning cycle.^3^ Analysis of the reported SE results ultimately in improvement measures which need to be implemented in order to improve safety. Not being able to complete this learning cycle will have an impact on patient safety and the possible recurrence of similar SE. Research suggests that the lagging improvement of patient safety is at least in part due to the quality of the improvement recommendations proposed in SE analysis reports. A US study, examining 320 SE analysis reports, found that the most frequently proposed solutions were actions that were less likely to reduce the recurrence of events.^1 2^ An Australian study, analysing 227 SE analysis reports, concluded that only a small proportion of the recommendations were high quality.^4^ In a survey of Dutch hospital executives responsible for patient safety, the majority confirmed they are confronted with a reoccurrence of SEs and indicated this might be partly due to the strength of recommendations.^3^ In these studies, the ‘strength’ or ‘quality’ of a recommendation is defined as its potential impact on reducing the risk or limiting the consequences of a similar SE in the future. This definition we will use in the remainder of this manuscript. In the learning cycle to improve quality and safety after an SE, every single step is essential.^3^ Failure to perform an adequate incident analysis, failure to compose high-quality recommendations or failure to implement these recommendations will be reflected in a much higher risk of reoccurrence of the SE. Methods for rational grading of the quality of recommendations after SEs into high-quality or low-quality categories are lacking.^5^

In a previous study, we developed and validated a basic set of criteria to determine whether recommendations found in SE analysis reports have the potential to prevent similar SEs. This was done because many of the derived recommendations from incident reports had no relation to the described SE or were formulated in such general terms that it was impossible to determine what was intended to or would change in practice.

These criteria are as follows: (1) a recommendation needs to be well defined and clear, (2) it needs to specifically describe the intended changes and (3) it needs to describe how it will reduce the risk or limit the consequences of a similar SE.^4 6^ The majority of recommendations proposed in current SE analysis reports do not meet these three basic criteria. The current study is a follow-up to this previous study. Ideally, only recommendations—which hold the potential to reduce the risk or limit the consequences of a similar SE— should be formulated by the team analysing an SE. Developing a method to grade recommendations could help in both designing and selecting those interventions most likely to have a positive impact on future healthcare safety. This method should be user-friendly, and grade recommendations based on objective features.^5^

In every healthcare organisation, choices have to be made since resources are limited. An objective validated method which can provide solid data for the dialogue on where to allocate resources, how risks should be weighed and prioritised and which interventions are proportional, is of the utmost importance. It makes more sense to implement a single extremely strong recommendation than several weak ones with a very limited potential to reduce risk.

**Figure 1:**
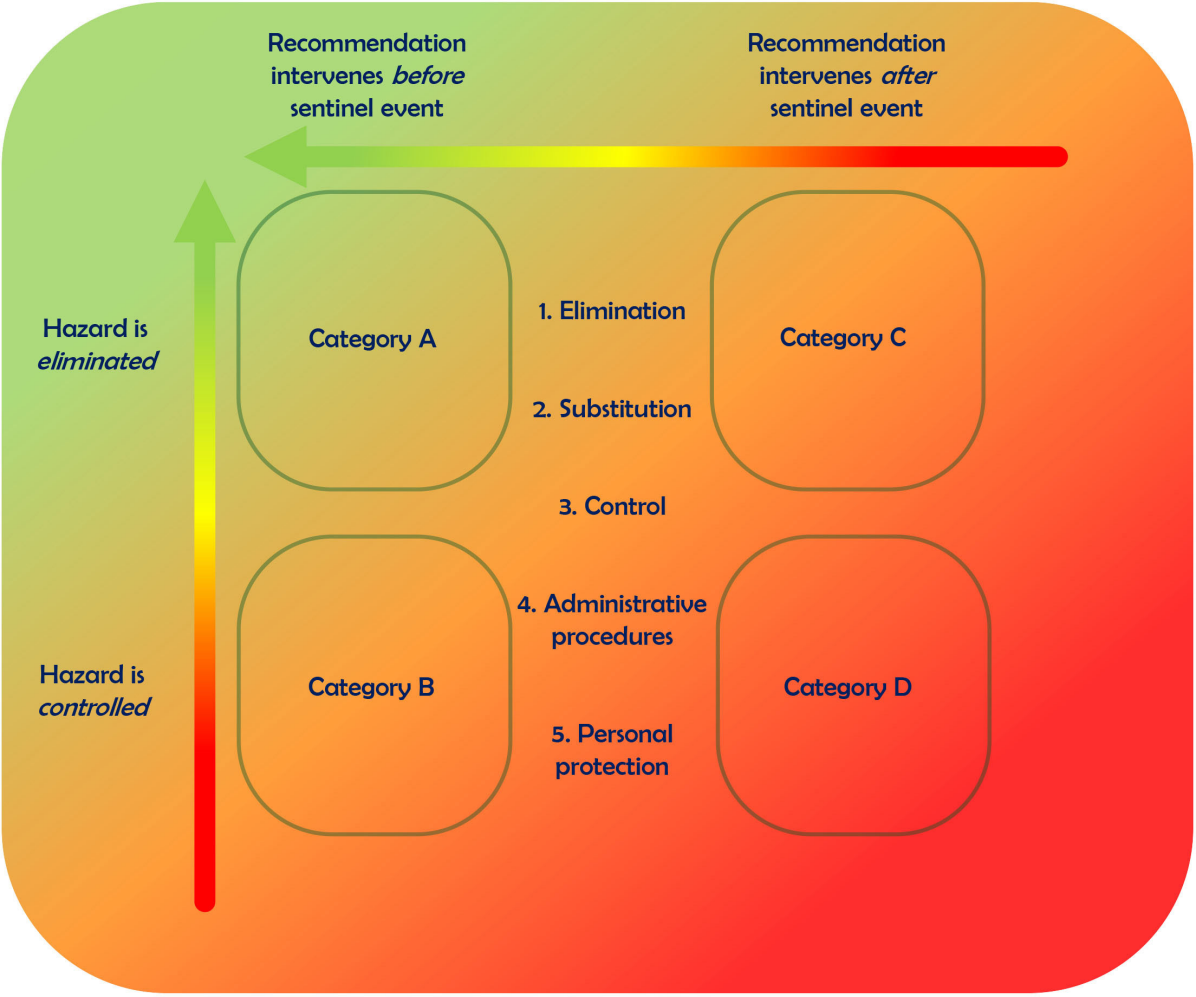
The recommendation improvement matrix.

Moreover by assessing applicability, that is, the degree to which the method is suitable for the purpose described above we determine if the recommendation improvement matrix (RIM) (figure 1) can be successfully used in real-world scenarios.

The aim of this study was to (1) develop a user-friendly method to grade the quality of recommendations in SE analysis reports and (2) assess the applicability and validity of this method in recommendations from analysis reports.

## Methods

A study design using multiple steps to develop and validate a grading method for recommendations after SEs was used. The first step was to create a grading method. After this, we stepwise tested and validated the method.

### Patient and public involvement

Patients or the public were not involved in the design, or conduct, or reporting, or dissemination plans of our research.

**Figure 2:**
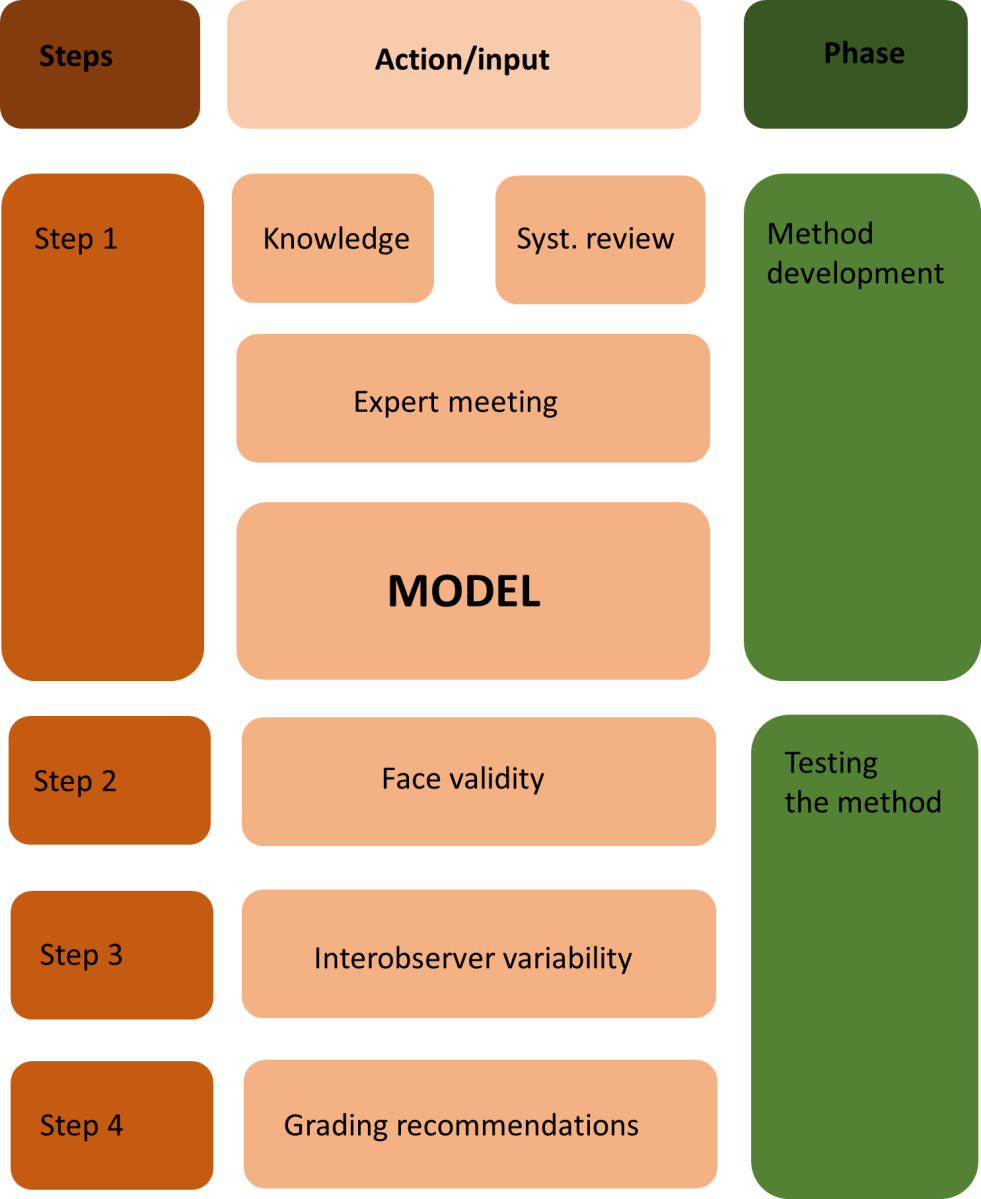
The flow chart on steps of the study.

Figure 2 overviews of the steps, with the inputs as well as the phases of the development of the RIM.

### Step 1: construct of the method

The goal of this step was to construct a method to grade recommendations after analyses of SEs. This was done by using the results of a previous performed systematic review and the input of team of experts. We organised a 4-hour live expert meeting with three medical doctors experienced in dealing with and analysing in-hospital SEs (in total approximately 20 years). Notably one of them was member of the hospital-wide committee responsible for the organisation of the analysis SEs, and two experts (an organisational psychologist with 3 years of experience and a safety engineer with over 20 years of experience in the safety industry) specialised safety in general and more specific human factors in safety-critical industries. The field of human factors involves the study of the interaction between humans and the environment in which they live and work.^6 7^ The purpose of the expert meeting was to develop a grading method for recommendations in SE analysis reports. In this expert consensus meeting, the data from a previous systematic review were used as the basis on which an objective dialogue in which a weighing and prioritising of these data was performed.^5^ The following characteristics of the included papers were discussed: user-friendliness, was the method validated, was the method aimed at recommendations after SEs and was the method objective.

### Step 2: face validity

The goal of this step was to test the face validity of the method. We asked four senior healthcare inspectors to use the method after a brief explanation. The Dutch Health and Youth Care Inspectorate (DHI) receives notifications on all SEs that occur in Dutch healthcare organisations. Following the hospital’s own analysis of an SE and the formulation of recommendations, a report is forwarded to the DHI. The DHI then assesses the quality of the reports, awarding a score between 0% and 100%.^8 9^ When the report receives an adequate score on all aspects (eg, analysis and recommendations), sometimes after revision by the hospital, the finalised report and all correspondence between the DHI and hospital is stored in the DHI database.^4^ The RIM was presented to four senior inspectors of the DHI specialised in assessing in-hospital SE analysis reports. This was the maximum number of inspectors available at the time. They were asked if the method could help grade the recommendations, that is, was it measuring the quality of recommendations after SEs and if they felt the method was user-friendly.

### Step 3: interobserver agreement

The goal of this step was to measure the interobserver agreement,^10^ this is done by using the results of 3 medical doctors’ rating and 16 recommendation using the grading method. To study interobserver agreement, we used 16 recommendations found in 10 randomly selected in-hospital SE analysis reports approved by the DHI between October 2017 and January 2018 (this time frame was chosen because the SEs had to be closed cases). After a short briefing, the recommendations were presented to three medical doctors experienced in dealing with in-hospital SEs and the aforementioned four DHI inspectors. All seven experts individually graded the recommendations using the RIM, after which interobserver agreement was tested. The process was carried out by individually comparing the placement of each recommendation within the matrix quadrants by each rater. It is important to note that no discussion occurred during the rating.

### Step 4: grading recommendations found in Dutch perioperative SE analysis reports

The goal of this step was to assess applicability, we graded the recommendations using the RIM. All perioperative SE analysis reports between July 2017 and July 2018 were selected in the DHI database. The selection of the category of perioperative SE was done on the basis of the fact that SEs in this setting reoccur (retention of a foreign material, wrong side surgery). The definition of a perioperative SE was an event that occurred prior, during or after the execution of a surgical procedure in the operating theatre by a medical doctor and an anaesthesiologist. The recommendations, outcome of the SE, hospital type and analysis period were extracted. Two researchers used the RIM (KB and A-FT (acknowledgements)) to grade the recommendations. In case of disagreement, consensus was reached through a short discussion.

### Statistical analysis

Interobserver agreement was tested by calculating the intraclass correlation coefficients (ICCs) and weighted kappa’s for the four categories (ie, A–D), including 95% CIs. Agreement for the 12 subcategories (A1–D5) was described. Values ≤0 were interpreted as indicating no agreement, 0.01–0.20 as none to slight agreement, 0.21–0.40 as fair agreement, 0.41–0.60 as moderate agreement, 0.61–0.80 as substantial agreement and 0.81–1.00 as almost perfect agreement.^10^ Categorical variables were presented as numbers (n) and percentages (%). IBM SPSS Statistics for Windows (V.26.0, IBM) was used to perform statistical analysis.

## Results

### Step 1: construct of the method

Although the methods identified in the review and discussed during the expert meeting were comprehensive, none completely satisfied our predefined criteria for an ideal recommendation prioritisation method. This gap led to the development of our own matrix, which aimed to synthesise the best features of the existing methods while addressing their limitations. In the expert meeting, it became clear that the method should make use of two axes, a time axis and a quality axis. In the time axis, it is determined if a recommendation intervenes before or after a specific SE. Subsequently, the quality of the recommendation is determined. The combination of both axis grades the recommendation on objective features (figure 2) and makes a grading or ranking of recommendations possible. Recommendations aimed at preventing the event are ‘stronger’ than those aimed at mitigating the effects after the event has occurred. This was derived from the so-called bowtie method which distinguishes barriers aimed to reduce the risk or limit the consequences of a similar SE.^11^ The bowtie method was thus used as a source for the time axis in the matrix. For the quality axis, a method described by McCaughan was selected.^12^ This model has been around for many years and is a standardised model in the occupational health space. The National Institute for Occupational Safety and Health, the USA, has long promoted this hierarchy^1 13^ It uses five clear and well-organised categories (elimination, substitution, engineering controls, administrative procedures and work practice controls),^12^ allowing the strength of each recommendation to be determined at a glance. These two methods combined were used to develop a grading matrix as shown in figure 2, which we hereafter refer to as the RIM.

The RIM method has two axes, as shown in figure 2.

1. The first step is to determine whether a recommendation aims to prevent an SE or to limit its consequences, that is, if the recommendation intervenes before or after an SE occurs.
2. The second step is to determine if the recommendation eliminates or controls the hazard.
3. The third and last step is to consider the subcategories. The subcategories all aim to prevent (left side of the RIM) the SE or limit its consequences (right side of the RIM). For example, if the SE was ‘a patient died following an unintended high dose of medicine X’, recommendations could be categorised as follows:

Elimination: Recommendations to eliminate the hazard.

For example, on the left side: ‘medicine X will no longer be available within the hospital’.

For example. on the right side: ‘an antidote that prevents death under all circumstances should be stored near the patient receiving medicine X’.

Substitution: Recommendations to substitute the hazard.

For example, on the left side: ‘medicine X is substituted by medicine Y that has similar effects but less dangerous side effects’.

For example, on the right side: ‘an antidote that reduces the risk of death should be stored near the patient receiving medicine X’.

Control: Recommendations for technical or physical interventions to eliminate (A/C) or control (B/D) the hazard.

For example, technical control on the left side: ‘medicine X is still available, but any attempt to prescribe it triggers a warning in the electronic prescription environment that highlights the risks, perhaps leading to cancellation of prescription’

For example, technical control on the right side: ‘a patient receiving medicine X is monitored by a computer that detects a change in vital functions and automatically administers the antidote if the patient has a tachycardia’.

Administrative procedures: Recommendations to control the hazard by adjusting procedures.

For example, on the right side: ‘when according to the protocol a patient is periodically checked by a nurse and the nurse is expected to administer the antidote should a patient experience a tachycardia’.

Personal protection: Recommendations to control the hazard by personal protection.

For example, on the right side: ‘a patient receives medicine X and the antidote is nearby, with the patient given responsibility for timely use of the antidote when experiencing a tachycardia’.

The subcategory control can exist in both the upper and lower quadrant, so a distinction can be made between stronger technical recommendations and weaker non-technical recommendations.

In case the SE was ‘a patient died following an unintended high dose of medicine X’, a recommendation could be introducing an antidote that limits the consequences, that fits category D. This means it potentially fits the subcategories control, administrative procedures and personal protection, depending on how the antidote is made available. If a patient receives medicine X and the antidote is nearby, with the patient given responsibility for timely use of the antidote when experiencing a change in vital functions, it fits the subcategory personal protection. When a patient is periodically checked by a nurse (procedure adjustment) and the nurse is expected to administer the antidote should a patient experience a change in vital functions, this is a non-technical recommendation that fits the subcategory administrative procedures. When a nurse automatically receives a signal about a change in vital functions in a monitored patient and the nurse is expected to administer the antidote, this is a partly technical recommendation that fits the subcategory control. When a patient is monitored by a computer that detects a change in vital functions and automatically administers the antidote, this is a technical recommendation that fits category C.

### Testing the method

#### Step 2: face validity

All four inspectors judged that the RIM is user-friendly, understandable and does not take much time. More importantly they found it potentially useful not only in their own practice but also as a tool for analysing teams to grade their own recommendations.

### Interobserver agreement

To asses interobserver agreement, 16 recommendations from in 10 randomly selected in-hospital SE analysis reports were graded by seven experts. The ICC was 0.38 (95% CI 0.20 to 0.63), indicating fair interobserver agreement. The mean weighted kappa was 0.37 and comparable to the ICC. More information on how the recommendations were graded can be found in (online supplemental file 1).

10.1136/bmjoq-2023-002592.supp1 Supplementary data

#### Step 3: grading recommendations found in Dutch perioperative SE analysis reports

To examine the potential applicability of the RIM, we graded recommendations from 115 perioperative SE analysis reports completed between July 2017 and July 2018. Of these SEs, 66% led to major injury and 34% resulted in death, with a majority (74%) occurring in the operating theatre. Of the 115 perioperative SE analysis reports, they offered a total of 442 recommendations, with only 161 meeting the three fundamental criteria previously discussed. Of these 161 recommendations, 44% were categorised as category A, 35% as category B, 2% as category C and 19% as category D, as shown in table 1.

**Table 1:** Number (n) and percentage (%) for the strength of recommendations found in Dutch perioperative sentinel event analysis reports between July 2017 and July 2018

Category A, n (%) Elimination, n (%) Substitution, n (%) Control, n (%)	70 (44) 3 (2) 62 (39) 5 (3)	Category C, n (%) Elimination, n (%) Substitution, n (%) Control, n (%)	4 (2) 0 (0) 3 (2) 1 (0)
Category B, n (%) Control, n (%) Administrative procedures, n (%) Personal protection, n (%)	57 (35) 3 (2) 54 (33) 0 (0)	Category D, n (%) Control, n (%) Administrative procedures, n (%) Personal protection, n (%)	30 (19) 1 (1) 29 (18) 0 (0)

## Discussion

In this study, the RIM was developed through multiple-step process. The RIM was designed to offer a grading method for recommendations after SE analysis. The grading system was created to be a user-friendly and objective method. Thereby it supports an essential step in the learning cycle necessary for improving quality and safety in healthcare.^3^ Creating effective recommendations from the SE analysis is a key step in this learning cycle. In a previous study, we defined that recommendations must meet three fundamental criteria if they are to have an effect on reducing the recurrence of similar SEs: (1) be well defined and clear, (2) specifically describe the intended changes and (3) describe how it will reduce the risk or limit the consequences of a similar SE in the future.^4 6^ The three criteria ensure that a recommendation has the potential to prevent similar SEs from recurring. The RIM offers insight into how the recommended intervention will affect the possibility of reoccurrence and thus patient safety: before or after the event, and by eliminating or controlling the hazard. Importantly by doing so, it supports the dialogue about the risk evaluation and considerations originating from SE analysis.

In a previous evaluation, we determined that just over one-third of the recommendations after SE had any potential to influence reoccurrence of that SE.^4 6^ Approximately, two-thirds were no effective recommendations that is, on the basis of the three previous described criteria they were dismissed. Recommendations like reconfirming operating procedures, email reminder to pay attention or discuss the case in the team, are obviously to general and non-specific as well as non-sustainable to have any impact on patient safety. The three described criteria support creating a recommendation that will affect the specific SE. The subsequent step involves creating and selecting the most robust recommendations that align with the nature of the SE. The strongest recommendation is in some cases not feasible or too expensive. To make weighted decisions and prioritise effectively, a method for grading recommendations is essential. The RIM supports this process, aiding organisations in optimally using their resources. The primary objective of the RIM is to facilitate informed and meaningful dialogue about recommendations, leading to the rational selection of the most effective measures to prevent the recurrence of an SE.

The strength of a recommendation is not the only parameter that predicts the potential impact of an SE analysis. Effects of this type of learning processes are often multifactorial.^3 9 14^ The recommendation is just one step in the path to improve patient safety. Previous research suggests more time and effort should be invested in implementing recommendations and monitoring their effect. However, effective implementation of ineffective safety recommendations will be of little value to patients or staff. We expect using the two-stage process, use the selection criteria and grade the recommendations with the RIM, will help identify the recommendations worthwhile of implementation.

The RIM has four categories, A–D, with decreasing potential to prevent harm. However, we value the RIM as a tool to help make informed decisions on which improvement measure best fits a particular situation. Of the 161 recommendations, we graded only 2% in category A1 which has the highest potential to reduce harm as these recommendations aim to eliminate the cause of harm before it occurs. This finding aligns roughly with an Australian study (8%).^4^ Although category A1 represents the strongest type of recommendation, it may not always be the most appropriate for every situation. For instance, in one case where an SE occurred due to an incorrect operation procedure, the hospital’s decision to cease performing that specific operation was a strong measure. While this could be a prudent decision in one context, it might deprive future patients of an effective treatment in another. We consider the RIM as a grading system that stimulates and supports constructive dialogue about what recommendations are the highest achievable recommendations possible. Then a risk assessment can be made in which all possible recommendations can be considered some can be selected for implementation. For example, a healthy young boy underwent an adenotonsillectomy (ATE) because of obstructive sleep apnoea syndrome. He was operated on as customary with short deep anaesthesia without securing the airway by intubation. He developed a haemorrhage which took a few minutes to control. A clot blocked his airway and he desaturated significantly during surgery. He had to be resuscitated, intubated and transferred to a paediatric intensive care unit. Securing the airway at all times during ATEs prevents an endangered airway and would be a recommendation in category A1, elimination. A crew resource management-training to ensure teams executing ATEs know how to adequately handle in case of an endangered airway before it leads to a significant desaturation would be a non-technical recommendation in category B, control. An emergency intervention team that can be called if the team executing ATEs cannot secure the airway before it leads to a significant desaturation would be a non-technical recommendation in category D, control. After exploring the possible recommendations with the RIM, those involved in formulating and/or selecting recommendations for implementation can have a structured dialogue to assess the risks within the healthcare organisation. The risk of a postoperative bleeding in ATE is low.^15^ If the airway is not secured, more children could undergo an ATE as they will not be needing securing the airway using an airway tube. Training the teams executing ATEs on how to adequately handle in case of an endangered airway is an option. Securing the airway at all times would prevent this SE to reoccur in the future. Those responsible for patient safety can now make an informed discussion on what risks are acceptable within the healthcare organisation. If the significant desaturation due to a bleeding in a healthy young boy is considered to be a low risk and the airway during ATE will not be secured in the future, similar SEs no longer need to be analysed as long as this risk will remain under the stated threshold. Fundamentally this structured dialogue based on the RIM within the organisation is about which risks are acceptable and which are not and what resources do a recommendation require to implement.

### Limitations

Current medical literature that focuses on learning from SEs in healthcare and formulating effective recommendations is limited. There is, however, there is an abundance of data from other high-risk, high-impact industries on safety improvement. During the expert meeting, we discussed literature identified during a previous systematic analysis, and the RIM was based on aspects of two of the methods found in that search.^11–13^ Data from other industries were included in our evaluation as well. This process was partly subjective as it involved a small panel of experts. There was no formal selection of the participants of the meeting this could have led to a selection bias. However, both methods used to construct the RIM come from other safety-critical industries in which the experts both worked. We tested the applicability of the RIM on recommendations found in Dutch perioperative SE analysis reports. Although we only found moderate interobserver agreement, in the absence of standards for the selection of recommendations the RIM is user-friendly, understandable and does not take much time. The RIM, or any alternative method, should not solely be seen as a tick-box or a set-in-stone truth on which recommendation is best. An important additional function is to better understand how recommendations are expected to work and enrich the dialogue between those responsible for improving patient safety. An important limitation of our study was the absence of a prospective analysis to further evaluate the outcome and applicability of the method. Future studies should, therefore, determine the effect of using the RIM on patient outcomes.

### Conclusion and future perspectives

In order to improve patient safety, a grading method for recommendations after SE is an essential next step. The RIM can be used to grade recommendations after SE analysis and support in both designing and selecting interventions. When combined with the three criteria for creating a recommendation (1) well defined and clear, (2) specifically describe the change and (3) how it will reduce the risk, the RIM is simple, user-friendly and has the potential to improve patient safety. While the RIM may help formulate effective and sustainable recommendations, a more important goal is to stimulate and support constructive dialogue between members of the analysing team when formulating and/or the head of the department or executive board when selecting recommendations for implementation. The RIM provides the essential data for a weighted decision of risks, resources and safety of processes in healthcare. By improving the dialogue of those responsible for patient safety, the RIM can help decrease the number of similar SEs. It needs to be kept in mind that creating the strongest recommendations possible is only one step in the learning cycle after SE. Reporting and analysing an SE as well as implementation of recommendations are essential steps as well. The full potential of the RIM’s use and impact may not yet be realised; its prospective application in other healthcare settings, including outside of hospitals, could unveil further implications. Subsequent research should explore the RIM’s applicability in prospective studies to definitively ascertain its effect on patient safety.

## Data Availability

All data relevant to the study are included in the article or uploaded as online supplemental information.
