# Supplementary material for: Grading recommendations for enhanced patient safety in sentinel event analysis: the recommendation improvement matrix
Source: BMJ Open Qual. 2024 Apr 16;13(2):e002592. doi: 10.1136/bmjoq-2023-002592 (PMC11029212; doi:10.1136/bmjoq-2023-002592)
Supplement: Supplementary data [file bmjoq-2023-002592supp001.pdf]

Supplementary file 1. Grading of the recommendations

A 1	C 1
2 IIII	2
3	3
B 3 III	D 3
4	4
5	5

Recommendation 1

A 1	C 1
2	2
3	3
B 3	D 3
4 IIIII	4 II
5	5

Recommendation 2

A 1	C 1 I
2 III	2
3	3
B 3 I	D 3
4	4 II
5	5

Recommendation 3

A 1	C 1
2 I	2
3	3 I
B 3 I	D 3
4 I	4 II
5 I	5

Recommendation 4

A 1	C 1
2	2
3	3
B 3 I	D 3
4 IIIII	4
5 I	5

Recommendation 5

A 1 IIIIII	C 1
2	2
3	3
B 3 I	D 3
4	4
5	5

Recommendation 6

A 1	C 1
2	2
3	3
B 3	D 3
4 IIII	4 III
5	5

Recommendation 7

A 1 IIII	C 1
2	2
3	3
B 3 I	D 3
4 II	4
5	5

Recommendation 8

A 1 I	C 1
2 I	2
3	3
B 3 I	D 3
4 IIII	4
5	5

Recommendation 9

A 1	C 1
2 I	2
3	3
B 3 III	D 3
4 I	4 II
5	5

Recommendation 10

A 1	C 1
2 I	2
3	3
B 3 I	D 3 I
4 II	4 I
5 I	5

Recommendation 11

A 1	C 1
2	2
3	3
B 3 I	D 3
4 III	4 III
5	5

Recommendation 12

A 1	C 1
2	2
3	3
B 3	D 3
4 I	4 IIIIII
5	5

Recommendation 13

A 1	C 1
2 III	2
3	3
B 3	D 3
4 IIII	4
5	5

Recommendation 14

A 1 IIIIIII	C 1
2	2
3	3
B 3	D 3
4	4
5	5

Recommendation 15

A 1	C 1
2	2
3	3
B 3 I	D 3 II
4 IIII	4
5	5

Recommendation 16
